# P-2090. Exploring Unmet Healthcare Needs among Foreign-born and US-born Transgender Women by HIV Status in the United States in 2019-2020

**DOI:** 10.1093/ofid/ofaf695.2254

**Published:** 2026-01-11

**Authors:** Patrick Eustaquio, Evelyn Olansky, Susan Cha

**Affiliations:** Independent Researcher, Metro Manila, Philipines, Pasig City, National Capital Region, Philippines; Independent Researcher, Atlanta, GA, Atlanta, Georgia; Independent Researcher, Atlanta, GA, Atlanta, Georgia

## Abstract

**Background:**

The intersection of foreign-born (FB) status and HIV among transgender women (TW) presents public health challenges, especially unmet healthcare needs (UHN). FBTW often encounter barriers like immigration status, language difficulties, and institutional distrust, which may be compounded for those living with HIV. Understanding how FB status and HIV intersect with UHN can guide health equity policies tailored to FBTW.

**Figure:**
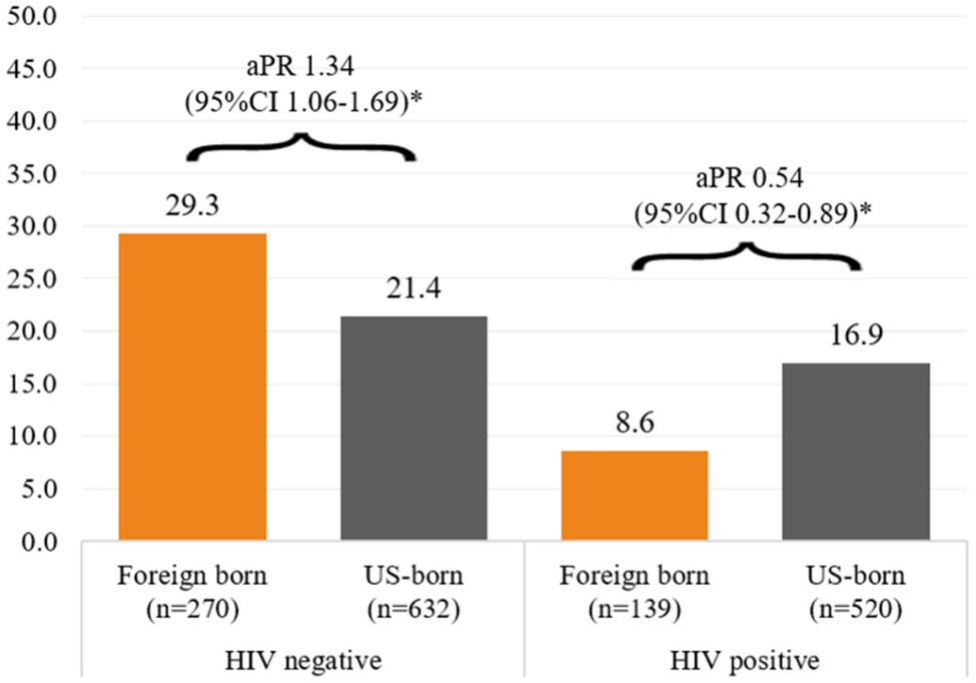
Prevalence of unmet healthcare needs among US-born and foreign-born transgender women by HIV status in 7 U.S. Cities from June 2019 – February 2020 Notes: aPR – adjusted prevalence ratios; CI – confidence intervals; * - significant at p<0.05, Models were adjusted for respondent-driven sampling design and confounding factors, including age and racial categories stratified by Hispanic/Latina ethnicity

**Methods:**

TW in 7 US cities were recruited via respondent-driven sampling, consented, interviewed, and offered HIV testing. Data from people who were ≥18 years, assigned male/intersex at birth, self-identified as women or TW, spoke English/Spanish, and who had valid responses to variables of interest were included. Participants were grouped by FB status: US-born vs. FB. Log-linked Poisson regression models estimated the association between FB status and past-year UHN due to cost. HIV test results and FB status were assessed for effect modification. Models generated adjusted prevalence ratios (aPR) and 95% confidence intervals (CI).

**Results:**

Among 1561 TW, 1152 (74%) were US-born and 409 (26%) were FB. 520 (42%) US-born TW and 139 (34%) FBTW tested HIV-positive. Overall, FBTW reported higher prevalence of UHN compared to US-born TW (22.2% vs 19.4%; aPR 1.15, 95%CI 1.01-1.46). There was a significant interaction between HIV test result and FB status (p< 0.05) (Figure). Among those HIV-negative, FBTW reported higher prevalence of UHN compared to US-born (29.3% vs 21.4%; aPR 1.34, 95%CI 1.06-1.69); whereas among those HIV-positive, FBTW reported lower prevalence of UHN’s compared to US-born (8.6%; vs 16.9% vs 8.6%; aPR 0.54, 95%CI 0.32-0.89).

**Conclusion:**

This finding challenge assumptions that HIV-positive FBTW, often at greater risk of comorbidities and requiring more medical care, would face more barriers to healthcare. Instead, HIV-negative FBTW having higher UHN’s may suggest lack of access to HIV prevention. This raises critical questions on underlying factors contributing to these UHN’s and suggests a complex interaction between HIV status and gender identity and its implications on healthcare access. Status-neutral, comprehensive care approaches must be prioritized for FBTW.

**Disclosures:**

All Authors: No reported disclosures

